# Health risk assessment of toxic elements and nitrate in tuberous vegetables: carcinogenic and non-carcinogenic perspectives, monte carlo simulation, and sensitivity analysis

**DOI:** 10.1038/s41598-025-23146-0

**Published:** 2025-11-10

**Authors:** Saeed Rajabi, Sobhan Maleky, Hassan Hashemi, Majid Hashemi, Majid Amiri Gharaghani, Hafez Shojaadini

**Affiliations:** 1 Environmental Health Engineering Research Center, Kerman University of Medical Sciences, Kerman, Iran; 2 Student Research Committee, Kerman University of Medical Sciences, Kerman, Iran; 3 Department of Environmental Health Engineering, Faculty of Public Health, Kerman University of Medical Sciences, Kerman, Iran; 4 Department of Environmental Health Engineering, School of Health, Jiroft University of Medical Sciences, Jiroft, Iran; 5 Research Center for Health Sciences, Institute of Health, Shiraz University of Medical Sciences, Shiraz, Iran; 6 Department of Environmental Health Engineering, School of Health, Shiraz University of Medical Sciences, Shiraz, Iran; 7 Student Research Committee, Shiraz University of Medical Sciences, Shiraz, Iran

**Keywords:** Carcinogenicity, Health risk assessment, Nitrate, Potential toxic elements, Sensitivity analysis, Cancer, Diseases, Environmental sciences, Risk factors

## Abstract

This study investigated the non-carcinogenic and carcinogenic risks associated with consuming carrots and radishes, focusing on heavy metal and nitrate contamination. Children had an overall hazard index (HI) for radish consumption of 23.77, in contrast to adult males (8.20) and adult females (9.49). The HI value for carrots was 38.81 in children; nevertheless, it was lower in adult males (10.46) and adult females (11.35). Zn had the lowest hazard quotient (HQ) values, ranging from 1.04 to 1.11; however, Cd and Pb showed the highest HQ values, especially in carrots (Cd: 13.05, Pb: 13.63 in children), hence presenting a more significant non-carcinogenic health risk compared to Zn and Ni. Geospatial analysis revealed Cd levels in radish samples ranging from 0.151 mg/kg to 4.096 mg/kg. In comparison, Pb levels ranged from 0.171 mg/kg to 6.794 mg/kg, exceeding the WHO’s permissible thresholds in some areas. However, Ni exhibited the highest carcinogenic risk among the analyzed metals, with values between 7.6 × 10^−6^ and 7.9 × 10^−6^, which, although below the USEPA criterion (1 × 10^−4^), were consistently above those of Cd and Pb. *Sobol* sensitivity analysis highlighted the importance of the intake rate, with a sensitivity index of 0.913, and Ni, with an index of 0.367, in assessing risks. The findings emphasized the need for targeted interventions like phytoremediation and stricter regulations to mitigate heavy metal exposure, especially for vulnerable populations like children.

## Introduction

Vegetables, as a significant source of dietary fiber, vital vitamins, minerals, and trace elements, are essential to the Iranian diet and many other countries, including India^1^, China^2^, and nations across the European Union^3^. As a consequence, vegetable safety is a concern that both the Iranian government and the people take very seriously^4,5^. Heavy metals known as potential toxic elements (PTE) are ubiquitous in the environment due to both natural and human-caused processes, and humans are exposed to them through different routes, the most common of which is the food chain^6–10^.

Zinc is an essential micronutrient, but too much might affect the immune system and copper metabolism. There are contradictory epidemiological findings on carcinogenicity^11^. In other studies, however, oxidative stress and the risk of prostate cancer have been associated with zinc homeostasis abnormalities^12,13^. Nickel compounds are categorized as a Group 1 carcinogen by the International Agency for Research on Cancer (IARC). Though occupational inhalation exposure is the primary route associated with nickel-associated carcinogenicity, nickel exposure may also occur through diet, smoking, coins, stainless steel, and jewelry in everyday living. Nickel intake through diet has been linked to dermatitis and gastrointestinal disorders. Also, some experimental and epidemiological studies suggest that nickel can cross the placenta and affect fetal growth and reproductive toxic effects, such as decreased fertility, spontaneous abortion, congenital malformations, intrauterine growth restriction, and low birthweight^14^. Epigenetic changes, DNA damage, apoptosis, and dysregulation of gene expression have been described as mechanisms of nickel toxicity. Collectively, this evidence points to nickel being a public health concern not only due to its established carcinogenic properties, but also regarding its negative impact on pregnancy and infant health outcomes^15–17^. Exposure to lead, mostly from contaminated food and water, is linked to several negative consequences, including neurodevelopmental deficits in children, renal toxicity, and hypertension, as well as possible stomach and lung cancers. Inorganic lead compounds are classified as possibly carcinogenic (Group 2 A) by the IARC^18^. Lead passes through the placenta, accumulates in fetal tissues, and has been associated with intrauterine growth restriction (IUGR), low birth weight, and neurodevelopmental effects. Even low levels of exposure can cause heme synthesis inhibition, renal dysfunction, and long-term cardiovascular effects. Overall, these studies highlight that lead constitutes a significant public health hazard, especially in pregnancy and early childhood^19–22^. Cadmium is known for its highly toxic nature. Chronic dietary cadmium exposure correlates with diminished renal function, bone demineralization, and heightened risk of prostate, lung, and endometrial cancers^23–28^. Cadmium is designated as a Group 1 carcinogen by the IARC^29^.

Nitrate is often considered harmless, but its conversion to nitrite in the stomach may potentially interact with amines, leading to the production of nitrosamines. This molecule, which is known to cause cancer, may also lead to gastrointestinal cancer, as well as teratogenic methemoglobinemia and mutagenic substances^6,30^. The absorption of dietary nitrate mostly occurs via ingesting vegetables and fruits, accounting for around 80% of the intake. Factors such as genetics, environmental conditions, light-to-soil ratio, soil type, ambient temperature, humidity level, other plant species in the field, plant growth degree, crop yield collection time, storage duration, and nitrogen source can all influence nitrate levels and their accumulation in fruits and vegetables^31,32^.

In recent times, probabilistic health risk assessments have become favored over traditional approaches to measure food safety, especially concerning toxic substances^9^. Health risk assessment is an invaluable technique for assessing the possible adverse health impacts of human exposure to environmental risks. It facilitates making decisions and implementing corrective measures to manage the quality of ingested vegetables. In addition, the HRA assists in monitoring and comprehending the air, water, and environmental quality, guiding individuals to prevent any hazards^33,34^. Nevertheless, uncertainty in HRA is inevitable because of inadequate and inaccurate data, measurement mistakes, and other constraints in information^35,36^. Usually, deterministic or point-estimating methods are used to assess the risk level of pollutants at a specific location. To minimize ambiguities and inaccuracies, risk levels may be evaluated using probabilistic methods that use probability distribution functions (PDFs)^37,38^. This approach allows for the measurement of risk levels, offering a thorough evaluation of risk that considers uncertainty and establishes the upper and lower limits of the actual risk value^39^.

The concentrations of nitrate and heavy metals (Pb, Cd, Zn, and Ni) in tuberous vegetables (carrot and radish) vary among Shiraz City’s districts and present quantifiable non-carcinogenic and carcinogenic health concerns to the local populace. This research aimed to directly evaluate the potential health risks posed to the general population by consuming tuberous vegetables such as carrots and radishes. For this research, the amounts of heavy metals of lead, cadmium, zinc, and nickel found in vegetables were measured. Following that, the overall health hazards associated with nitrate were assessed for the general population in Shiraz Metropolitan. Finally, a *Sobol* sensitivity analysis was carried out to identify the influence of the respective factors.

## Materials and methods

### Study area

**Fig. 1:**
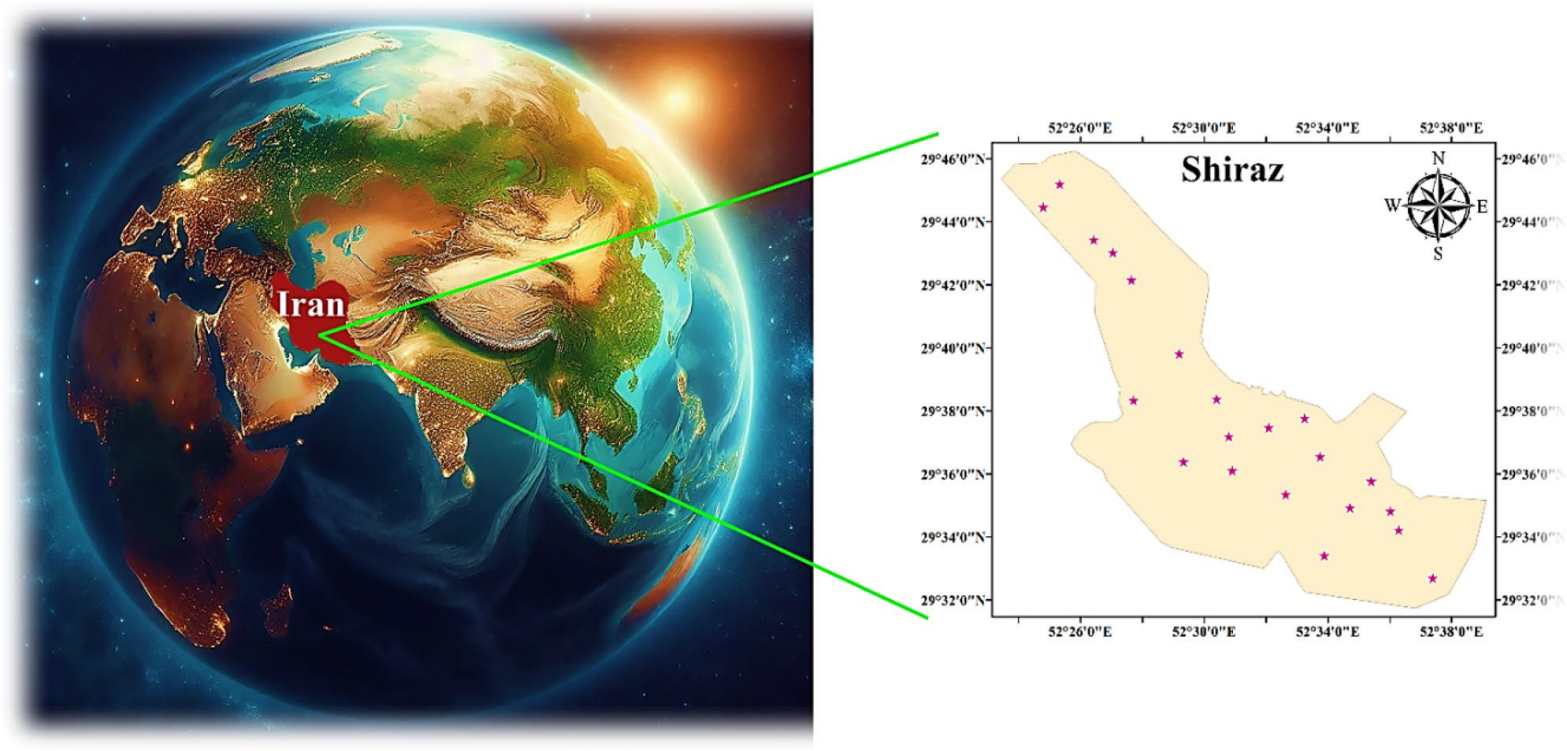
Study area and sampling sites.

The southern Iranian province of Fars is home to the capital city of Shiraz, which is located at 52°32′ E and 29°37′ N. Asmari and Razak are the two geological formations in Shiraz characterized by sedimentary formations primarily consisting of marl, limestone, and gypsum. Calcareous soils with a high pH, moderate organic matter, and varying clay content are the most common soil types in the area. These soil types can impact the mobility and bioavailability of heavy metals. Carbonate and phosphate minerals in certain soils may bind metals like lead and cadmium, influencing how well plants absorb them^40^. Most of the Razak structures are located in urban and residential areas. On the north and northwest edges of the Shiraz Plain are the mountains Drak, Bamu, Baba Koohi, and Poshte Mole. Figure 1 illustrates the areas of investigation. The annual mean temperature is 18℃. With an average height of 1480 m above sea level, the region receives around 337 mm of rainfall yearly. Shiraz’s climate is generally classified as a hot semi-arid zone according to the Köppen climate categorization. Shiraz is traversed by the seasonal Dry River. Upon crossing the city, the watercourse proceeds in a southeastern direction towards its drainage basin, ultimately discharging into Maharloo Lake^41^.

### Sample preparation

Samples of the tuberous plants, including radish and carrot, were gathered from 21 sites from 11 central districts within the Shiraz metropolitan area, and labeled polyethylene sample bags were used for storage under chilled conditions. The selection of sampling sites was influenced by several factors, including crop diversity, agricultural intensity, and proximity to potential sources of contamination, such as industrial zones, urban runoff, and areas irrigated with wastewater or groundwater known to have elevated levels of heavy metals. These sites also represented sites with significant local vegetable production. Various land-use types, such as residential, peri-urban, and agricultural zones, were included in the sampling districts chosen to ensure representativeness of exposure scenarios and broad geographical coverage. Subsequently, the consumable portions were divided. Ultimately, it is rinsed with tap water to eliminate any residues, such as dirt particles. Afterward, the consumable portions of the vegetables were dehydrated in the oven and transformed into a fine powder to facilitate the digestion of the plant. A 0.5 g sample of dried vegetables was placed in a Pyrex container. A solution containing about 15 mL of a combination of HNO_3_, HClO_4_, and H_2_SO_4_ in a ratio of 5:1:1 was poured into the beaker and allowed to sit undisturbed at room temperature for a night. The digesting tubes were then subjected to a temperature of 80 °C for 60 min, followed by a steady rise to 120–130 °C until a transparent solution was achieved. After digestion, the samples underwent filtration, and the volume was increased to 50 mL by adding deionized water^42,43^. The sample preparation and extraction processes for nitrate measurement were conducted under Food Standards 16,721–2. Nitrate evaluations were carried out using ion chromatography. 10 g of dried vegetable powder was poured into a volumetric flask with 400 ml of boiling water and let stand in a Bain-Marie for 15 min. Following room temperature cooling, Whatman quantitative filter paper Grade 41 was used to filter the mixture^44^.

### Heavy metals and nitrate measurement

A flame atomic absorption spectrophotometry (FAAS) with a GBC SavantAA model was used to analyze the samples of digested vegetables for the presence of heavy metals such as Zn, Ni, Cd, and Pb according to the Standard Reference Materials (SRMs)^45^. To determine the analytes, the wavelengths (nm) that were chosen were Cd 228.8 (LOD: 0.003 mg/kg, LOQ: 0.01 mg/kg), Ni 232.0 (LOD: 0.02 mg/kg, LOQ: 0.06 mg/kg), Pb 283.3 (LOD: 0.01 mg/kg, LOQ: 0.03 mg/kg), and Zn 213.9 (LOD: 0.01 mg/kg, LOQ: 0.03 mg/kg)^46^. By ion chromatography (Metrohm Compact IC Plus 882 Model from Switzerland), 40 mL of the filtered solution was analyzed to measure the nitrate content (LOD: 0.2 mg/L, LOQ: 0.6 mg/L). The interfering chemicals were eliminated using Carrez reagents. Dilute the final samples ten times and filter them with a 0.45 μm and 0.22 μm mixed cellulose ester (MCE) syringe filter to avoid damage to the ion chromatography column and decrease vegetable color interference^44^.

### Health risk assessment

#### Non-carcinogenic risk

To evaluate the health risks among three age and gender categories—children, adult men, and adult females—the United States Environmental Protection Agency (USEPA) developed a model. This model comprises three channels for pollutant translation: ingestion, dermal absorption, and inhalation. The present research investigated the method by which plants accumulate PTEs and nitrate via consumption. The Hazard Index (HI) approach was used to adhere to the recommendations for measuring health hazards. The HI technique has many advantages, including its extensive track record, compressibility, simplicity of implementation, and provision of assistance for health issues. An individual’s HI is the estimated prospective health risk due to the accumulation of their Hazard Quotient (HQ) from all chemical constituents to which they are exposed. To evaluate the non-carcinogenic risk associated with vegetable intake among the Shiraz population, Eq. 1 to 3 were utilized:

1 $$\:{{Intake}}_{{Oral}}{\:=\:}\frac{{ED} \times {\:C}\:\times \:{EF}\:\times \:{IR}}{{BW}\: \times {\:AT}}$$

2 $$\:{HQ\:=\:}\frac{{{Intake}}_{t{Oral}}}{{RfD}}$$

3 $${HI = }\sum{HQ}$$

The variable *C* indicates the pollutant concentration (mg/kg), whereas the variable *IR* indicates the intake rate (g/day.person). Exposure frequency (*EF*) refers to the number of times an event happens in a specific timeframe. Exposure duration (*ED*) indicates the length of exposure in years, *BW* refers to the average body weight in kg, *AT* designates the average duration of life expectancy in days, and *RfD* stands for the reference dosage in mg/kg.day. The USEPA’s Integrated Risk Information System (IRIS) database provides the following Reference Dose (*RfD*) values for Pb, Cd, Zn, Ni, and Nitrate: 0.00357, 0.001, 0.3, 0.02, and 1.6 mg/kg.day, respectively. Values of HQ or HI below 0.1 are deemed insignificant; values between 0.1 and 1 are considered to have a low significance level regarding health impacts. Values between 1 and 4 provide a moderate risk with considerable health effects, while values over 4 have a very high risk^47,48^.

#### Carcinogenic risk

Carcinogenic risk (CR) is a quantification of the additional lifetime occurrence of cancer that is used to indicate the likelihood of acquiring cancer when exposed to a certain amount of a cancer-causing substance. The slope factor is the numerical value assigned to this metric. The present research includes Cd, Pb, and Ni in the Integrated Risk Information System (IRIS), where they are assigned oral slope factors of 0.38, 0.0085, and 1.7, respectively. CR calculations were performed to determine the consumption of two distinct vegetables, namely Radish and Carrot, using Eq. 4:

4 $$\:CR\:=\:\frac{ED\:\times\:\:C\:\times\:\:EF\:\times\:\:IR}{BW\:\times\:\:AT}\:\times\:\:{S}_{F}\:\times\:\:{10}^{-3}$$

The equation parameters were considered as a non-carcinogenic risk equation. The CR formula values are shown in Table 1. Additionally, risks below 1 × 10^−6^ are not considered to pose a significant risk to health, risks between 1 × 10^−6^ and 1 × 10^−5^ are supposed to represent a low significant risk, risks between 1 × 10^−5^ and 1 × 10^−4^ are classified as medium-range, risks between 1 × 10^−4^ and 1 × 10^−3^ are determined to pose a high health effect, and risks above 1 × 10^−3^ are regarded to be relatively high^49,50^.

**Table 1:** Health risk assessment parameters’ values.

Factor	Unit	Definition	Value		
			Children	Adult Male	Adult Female
IR	Intake	kg. day^−1^	0.13	0.26	0.26
EF	day. year^−1^	Exposure frequency	365	365	365
ED	years	Exposure duration	6	30	30
BW	kg	Body weight	18.6	66.2	57.3
AT	days	Average time (Non-carcinogenic)	10,950	10,950	10,950
		Carcinogenic	25,550	25,550	25,550

### Statistical analysis

#### Spatial distribution

Geographic Information System (GIS) software was used to analyze the geographical distribution of Cd, Pb, Zn, Ni, and NO_3_ concentrations (ArcGIS v. 10.8.2). To interpolate the spatial distribution of these components over the research region, the Inverse Distance Weighting (IDW) deterministic approach was used. With the help of neighboring sampled sites’ values, weighted by the inverse of the distance, IDW, a deterministic method of spatial interpolation, calculates a variable’s value in unsampled places^51^.

#### *Sobol* sensitivity analysis

In this study, *Sobol* Sensitivity Analysis was combined with a Monte Carlo technique to identify key input variables and assess their impact on the variance of exposure results. This technique is necessary for assessing exposure risk under uncertain and variable conditions, which is a key consideration of real-world scenarios, where contaminant concentrations are dynamic^52^. *Sobol* Sensitivity Analysis operates on the principle of total variance (Eq. 8), which can be normalized as shown in Eq. 9. It decomposes a model function *f* (Eq. 5) into increasingly complex components^6,53,54^:

5 $$\:Y\hspace{0.17em}=\hspace{0.17em}f\left(X\right)\hspace{0.17em}=\hspace{0.17em}f\:(x1,\:x2,\:x3,\:\dots\:,\:xp)$$

*Y* represents the scalar prediction model, and *X* = (*x*_*1*_, *x*_*2*_, *x*_*3*_, …, *x*_*p*_) denotes the set of model input variables.

6 $$\:V\left(Y\right)\:=\sum\:_{i=1}^{p}{V}_{i}+\sum\:_{i=1}^{p-1}\sum\:_{j=i+1}^{p}{V}_{ij}+...+{V}_{1...p}$$

7 $$\:1\:=\sum\:_{i=1}^{p}{V}_{i}/V+\sum\:_{i=i}^{p-1}\sum\:_{j=i+1}^{p}{V}_{ij}/V+...+{V}_{1...p}/V$$

This framework compares single-factor variance (partial variable) with the total output variance (interactions among variables). Each term in Eq. (7) represents a variable up to the *p*_*th*_ order, with the *Sobol* Sensitivity Indices (SIs) being the ratio of partial variance to total variance. The First Order Sensitivity Index (*FOSI*) is the first term in Eq. (7) and measures the effect of an individual variable (*x*_*j*_) on the variance of model outputs (Eq. 8). The Second-Order Sensitivity Index (*SOSI*) is the second term, indicating the effect of the interaction between variables (*x*_*i*_) and (*x*_*j*_) (where *i* ≠ *j*) (Eq. 9).

8 $$\:FOSI\:Si\:=\:Vi/V$$

9 $$\:SOSI\:Sij\:=\:Vij/V$$

The Total Order Sensitivity Index (*TOSI*) calculates the overall effect of a variable on the variance, including all interactions, and is determined by the variance (*V*_*i*_) when all parameters except (*x*_*i*_) are varied (Eq. 10).

10 $$\:TOSI\:STi\:=\:Si\:+\sum\:_{j\ne\:i}{S}_{ij}+...=1-\left({V}_{i}/V\right)$$

Sensitivity indices are classified as highly sensitive (> 0.1), sensitive (0.01–0.1), or insensitive (< 0.01) based on their values^55,56^. The *Sobol* Sensitivity Analysis for this study was conducted using R-platform packages (‘*EnvStats*’, ‘*sensitivity*’, and ‘*EnviroPRA*’) version 4.4.

#### Monte carlo simulation

This research employed Oracle Crystal Ball software version 11.1.4716 to evaluate health risk using a probabilistic methodology. A Monte Carlo simulation with 50,000 iterations was used to assess the uncertainty and variability of the data thoroughly. The lognormal distribution was selected to simulate the distribution of input variables, since it is deemed appropriate for environmental data characterized by positive values and asymmetrical distribution. During this procedure, input values were generated randomly following the specified distribution parameters, followed by the computation of the associated risk indices. The use of several iterations enhanced the accuracy of the results and included a broad spectrum of potential circumstances^41^.

## Results and discussion

### Health risks of vegetable consumption

**Fig. 2:**
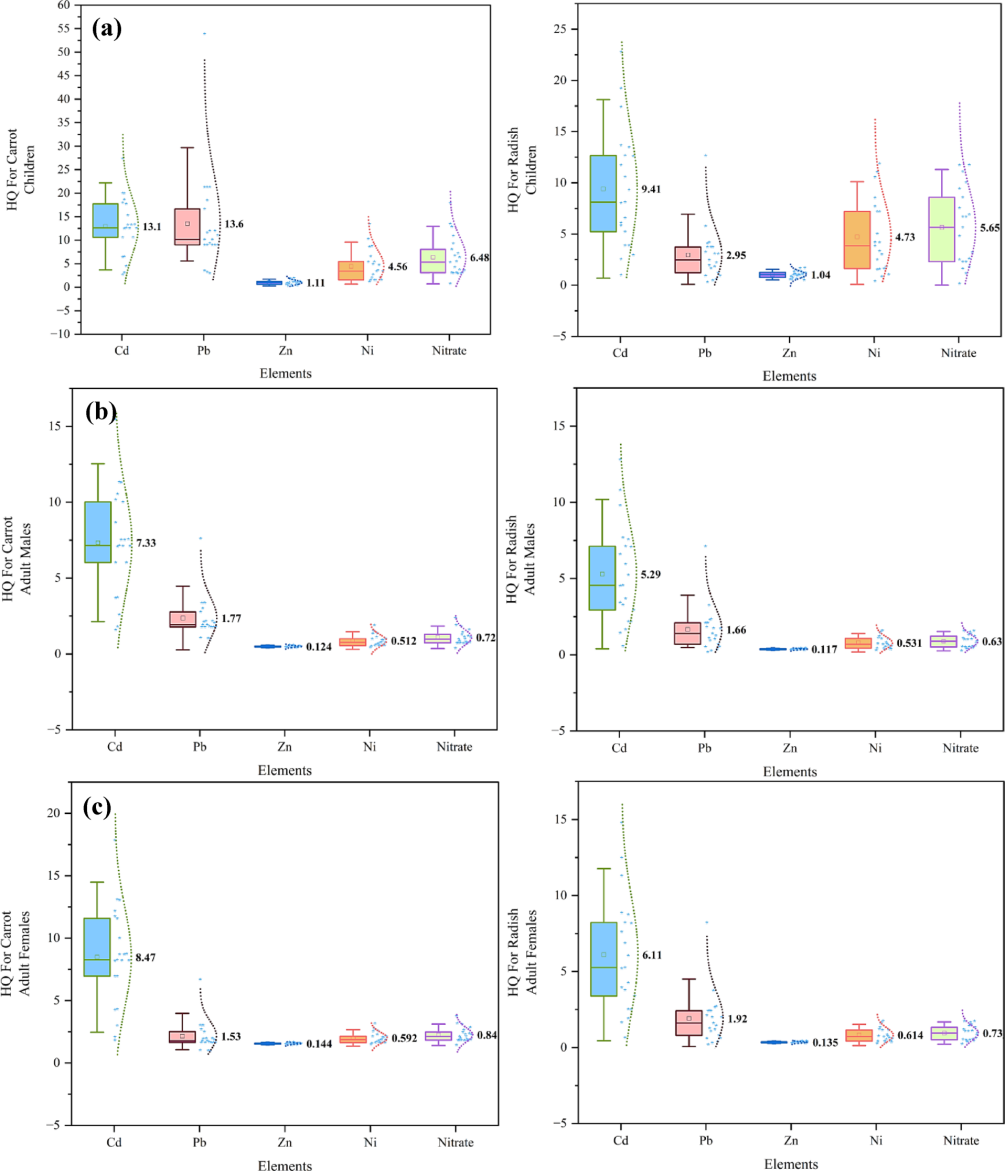
Non-carcinogenic health risk assessment for children (**a**), adult males (**b**), and adult females (**c**).

The HQ and HI values are shown in Table 2. Table 2 shows that the HQ values in children were consistently greater than those in adults, which aligned with earlier research^57–60^. Additionally, among the adult population, the levels of HQ and HI were somewhat elevated in females compared to men. The children’s HQ values for radish intake were ranked in the following order: Cd > Nitrate > Ni > Pb > Zn. Furthermore, the HQ values were ranked as Pb > Nitrate > Ni > Cd > Zn for carrot consumption in children. In addition, the HI and HQ values for ingesting radish and carrot were over 0.1 for children, adult men, and females (Fig. 2a-c). This indicated a degree of worry and high risk. The findings revealed that the carcinogenic risk associated with consuming carrots is higher than that of consuming radishes in children and adults of both genders. Among the three studied groups, the average carcinogenic risk values for Ni ranged between 7.06 × 10^−6^ and 7.93 × 10^−6^, which were below the USEPA threshold of 1 × 10^−4^, but consistently higher than those of Cd and Pb (Table 3).

#### Non-carcinogenic risk assessment

Based on the findings, the HQ values were consistently greater in children compared to adult men and women. The elevated values in children may be attributed to their body weight. Furthermore, Cd had the most outstanding non-carcinogenic risk (children’s HQ for radish = 9.41), while Zn had the lowest HQs (≈ 1.04–1.11). Ni exhibited the moderate HQ values (≈ 4.55–4.72 in children). Cd had a significant impact on the non-carcinogenic risk associated with consuming radishes. Even at low levels, it mainly accumulates in the kidneys, lungs, and liver^14^. These findings were consistent with prior research investigations^59,60^. A further investigation carried out in Kashan, Iran, revealed that the levels of Cd and Pd in radish were above the maximum permissible thresholds set by the World Health Organization^61^; furthermore, the findings from consuming carrots aligned with a comparable study^60^. Pb, Cd, and nitrate concentrations in carrot and radish are significantly affected by pH, particle size, soil cation exchange capacity, root exudation, and other physicochemical factors. Moreover, this phenomenon might be ascribed to excessive use of inorganic fertilizers or the existence of specific pesticides^62^. Moreover, elevated levels of Zn might be linked to the use of manure fertilizer in vegetable tissues. Furthermore, the amounts of Pb and Cd exceeded the criteria set by FAO and WHO, 0.3 and 0.2 mg/kg, respectively^63^. In addition, separate research conducted in Dhaka, Bangladesh, evaluated the non-carcinogenic risk associated with heavy metals in vegetables. The findings indicated that the HI value for radish was 0.362, indicating a minimal health risk associated with consuming it. Furthermore, while considering various kinds of vegetables that were examined, it was found that both carrots and radishes had a quantity of Cd (0.5 mg/kg) above the recommended standards set by the FAO/WHO^64^. Research was undertaken in the Rappur region of Pabna District in Bangladesh to evaluate the health risks associated with heavy metals in ingested vegetables. The computed HQ values for Pb, Ni, Zn, and Cd in the vegetables were 0.027, 0.194, 1.051, and 2.543, respectively. These results indicate that only Cd presents a moderate health concern^65^.

The human health risks associated with heavy metal pollution in vegetables were assessed in Bahawalpur, Pakistan. The results indicated that in adults, the HI values associated with Cd were 1.600 and 1.602 for radish and carrot, respectively. Additionally, the HI values for Cd in radish were determined to be 1.830 and 1.840 for children, as reported in reference^66^. Separate research examined the possible health hazards of heavy metals (such as arsenic, lead, and cadmium) when people regularly consume vegetables. The results showed that Pb has the greatest HQ compared to other heavy elements. Furthermore, the headquarters of females was found to be bigger than men’s^67^. The research examined the levels of Pd, Cd, Cr, and Ni in adult men and women from the Noakhali area in Bangladesh. The findings demonstrated that the THQ values of Pb and Cd in almost all vegetable species were over 1, suggesting that consuming these vegetables might provide a non-carcinogenic danger^57^. A separate investigation in Arequipa, Peru, examined the health hazards of consuming vegetables contaminated with heavy metals (Hg, Cd, Cr, and Pb). The research revealed that the HQ values for children were higher than those for adults, and Pb had a higher HQ than Cd. Additionally, Pb exhibited a greater HQ compared to Cd. Moreover, Cd had the most significant influence on the non-carcinogenic risk of vegetables^60^. A study was conducted in Hamedan, Iran, to examine the potential health risks of heavy metals in agricultural soil and food crops. A study was conducted to analyze the concentration of heavy metals in crops. The findings indicated that the HQ values of Zn, Ni, Cd, and Pb were relatively low (10^−1^), suggesting no significant danger to the population under study. Conversely, the values of children’s HQ were somewhat greater than those of adults. Furthermore, the order of the HQ was adhered to, with Pb having the highest value, followed by Cd, Zn, and Ni^58^.

**Table 2:** Obtained HQ for children, adult males, and adult females.

Vegetable	Element	Average HQ for Children	Average HQ for Adult Male	Average HQ for Adult Female
**Radish**	Cd	9.411 ± 5.81	5.288 ± 3.26	6.11 ± 3.77
	Pb	2.95 ± 2.66	1.65 ± 1.49	1.91 ± 1.72
	Zn	1.04 ± 0.341	0.11 ± 0.038	0.13 ± 0.04
	Ni	4.72 ± 3.58	0.53 ± 0.4	0.61 ± 0.46
	Nitrate	5.65 ± 3.76	0.63 ± 0.42	0.73 ± 0.48
	**HI**	**23.77 ± 16.14**	**8.2 ± 5.61**	**9.49 ± 6.47**
**Carrot**	Cd	13.05 ± 6.17	7.33 ± 3.47	8. 47 ± 4.01
	Pb	13.63 ± 10.76	1.77 ± 1.39	1.53 ± 1.2
	Zn	1.11 ± 0.48	0.124 ± 0.05	0.124 ± 0.062
	Ni	4.55 ± 3.43	0.512 ± 0.38	0.51 ± 0.44
	Nitrate	6.47 ± 4.39	0.72 ± 0.5	0.72 ± 0.47
	**HI**	**38.81 ± 25.23**	**10.456 ± 5.8**	**11.35 ± 6.18**

#### Carcinogenic risk assessment

**Fig. 3:**
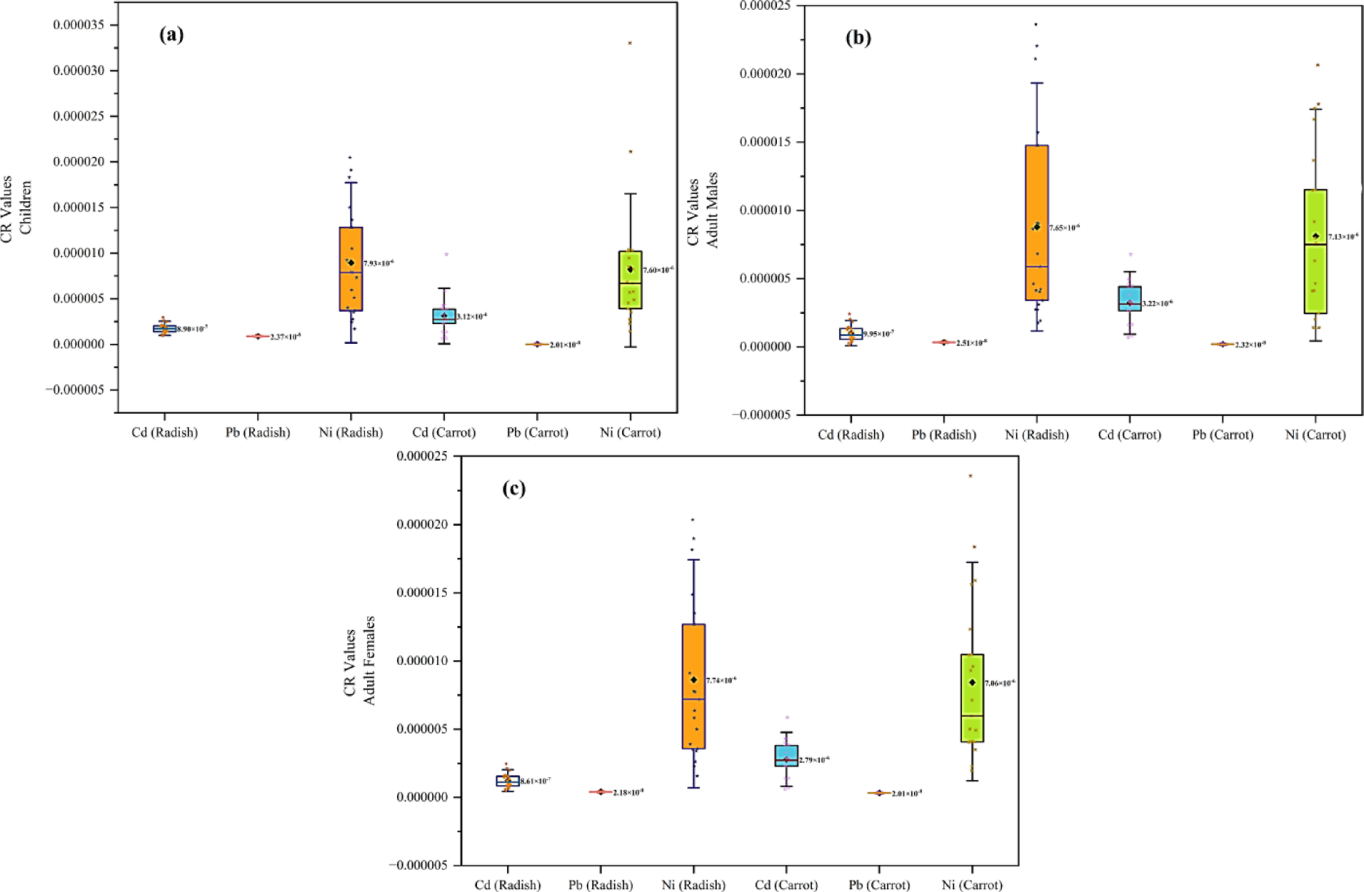
Carcinogenic health risk assessment for children (**a**), adult males (**b**), and adult females (**c**).

Exposure to heavy metals such as Pb, Cd, and Ni may increase the likelihood of developing cancer in people. Prolonged exposure to low concentrations of toxic metals may lead to the development of several types of cancers. The CR values of Cd and Pb were below the USEPA permissible level for risk, indicating no major risk concerns to any of the groups investigated (Fig. 3a-c). On the other hand, Ni showed CR values that were relatively higher: an average, for radish, of 7.93 × 10^−6^ (children), 7.65 × 10^−6^ (adult males), and 7.74 × 10^−6^ (adult females), and an average of 7.60 × 10^−6^ (children), 7.13 × 10^−6^ (adult males), and 7.06 × 10^−6^ (adult females), for carrot. While below the USEPA acceptable threshold, they were solidly higher. When looking across metals, the carcinogenic risk ranking was Ni > Cd > Pb for both radish and carrot consumption, and this was consistent across children, adult males, and adult females. Likewise, total carcinogenic risk (TCR) values were also below the threshold established by the USEPA, with total ranging from 8.84 × 10^−6^ (children), 8.67 × 10^−6^ (adult males), and 8.62 × 10^−6^ (adult females) for radish, and 10.74 × 10^−6^ (children), 10.37 × 10^−6^ (adult males), and 9.87 × 10^−6^ (adult females) for carrot consumption. Overall, despite remaining at acceptable levels indicated by the CR values, the relatively high contribution of Ni, particularly in children, is an area of concern meriting continued observation regarding the safety of vegetables in the study area^57^.

**Table 3:** Obtained CR for children, adult males, and adult females.

Vegetable	Element	Average CR for Children	Average CR for Adult Male	Average Cr for Adult Female
**Radish**	Cd	Cd	8.88 × 10^−7^ ± 1.71 × 10^−6^	9.95 × 10^−7^ ± 5.31 × 10^−7^
	Pb	Pb	2.36 × 10^−8^ ± 3.065 × 10^−8^	2.51 × 10^−8^ ± 1.96 × 10^−8^
	Ni	Ni	7.93 × 10^−6^ ± 1.02 × 10^−6^	7.65 × 10^−6^ ± 5.87 × 10^−6^
	**TCR**	**TCR**	**8.84 × 10**^**−6**^ **± 2.76 × 10**^**−6**^	**8.67 × 10**^**−6**^ **± 6.42 × 10**^**−6**^
**Carrot**	Cd	Cd	3.12 × 10^−6^ ± 2.83 × 10^−6^	3.22 × 10^−6^ ± 1.32 × 10^−7^
	Pb	Pb	2.01 × 10^−8^ ± 2.42 × 10^−8^	2.32 × 10^−8^ ± 1.59 × 10^−8^
	Ni	Ni	7.6 × 10^−6^ ± 2.83 × 10^−6^	7.13 × 10^−6^ ± 1.32 × 10^−7^
	**TCR**	**TCR**	**10.74 × 10**^**−6**^ **± 5.68 × 10**^**−6**^	**10.37 × 10**^**−6**^ **± 2.79 × 10**^**−7**^

### Spatial distribution of metals and nitrate

Cadmium, lead, zinc, and nickel are a few heavy metals that enter soil systems from anthropogenic sources such as mining, application of phosphate fertilizers, sewage sludge application, gas emissions from industries, and natural weathering of parent materials^68^. The complexation with organic matter, precipitation/dissolution, and adsorption/desorption processes that the metals go through once they are in the soil impact their bioavailability to plants. Several variables, such as soil pH, cation exchange capacity (CEC), organic matter, redox potential, and metal speciation, vary significantly in plants’ heavy metal absorption. Roots absorb, move, and dissolve more lead and cadmium with decreasing pH^69^. The root epidermis is responsible for taking up heavy metals either by active transport mechanisms or by simple diffusion, which are not very different from the respective mechanisms of essential nutrients. For example, Ca^2+^ channels allow cadmium to enter the plant cells^70^. While some metals are retained in the root system after absorption, others are carried along in the xylem to aerial organs, including culinary organs like leaves, stems, and fruits. Plants grown in contaminated soil can absorb heavy concentrations of heavy metals, and humans can ingest the metals directly. Recurrent consumption of such polluted foodstuffs might cause chronic exposure, especially in areas lacking good food safety policies. This route contributes a high percentage of non-occupational exposure to toxic metals, especially among vulnerable groups such as pregnant women and children^71^.

**Fig. 4:**
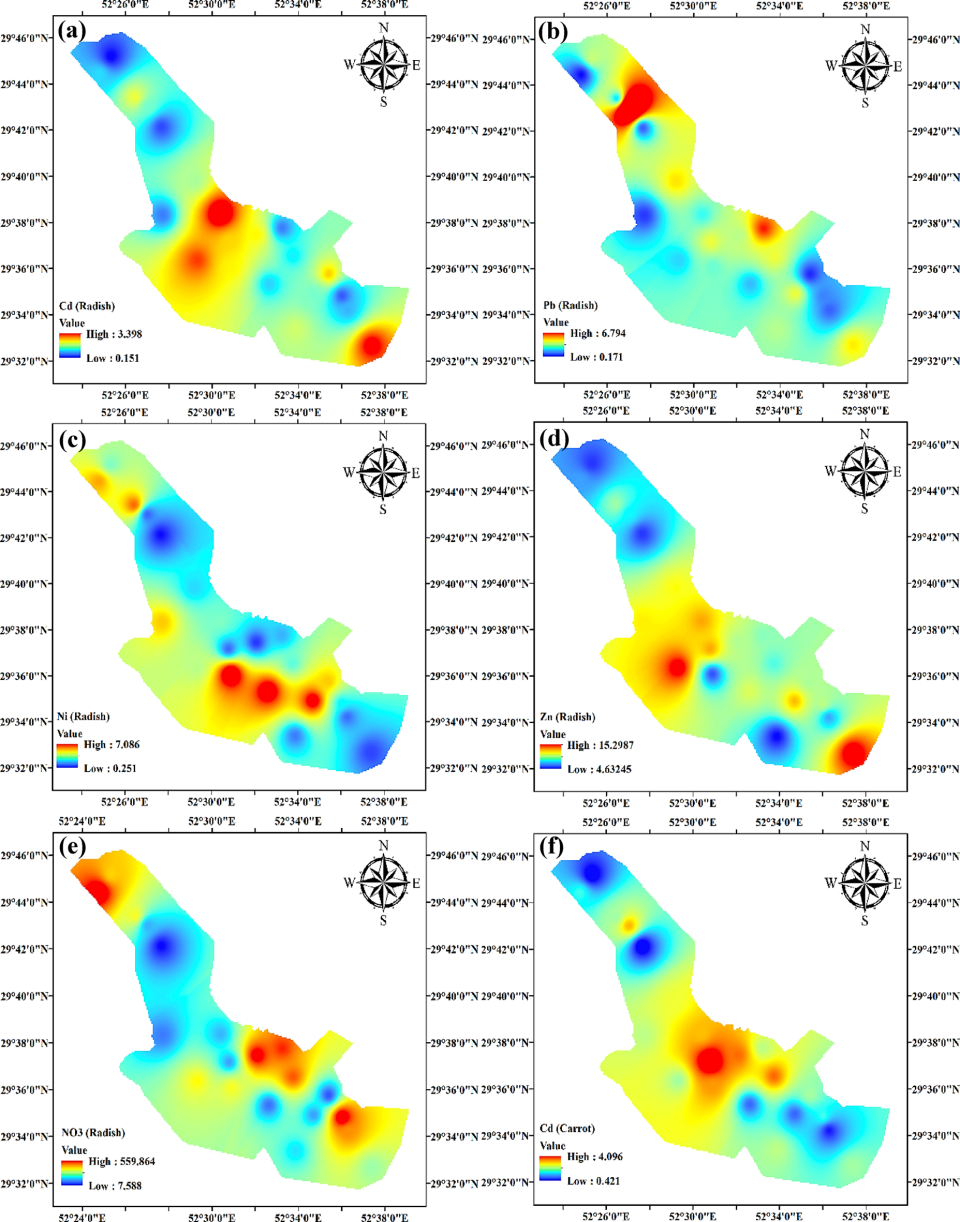

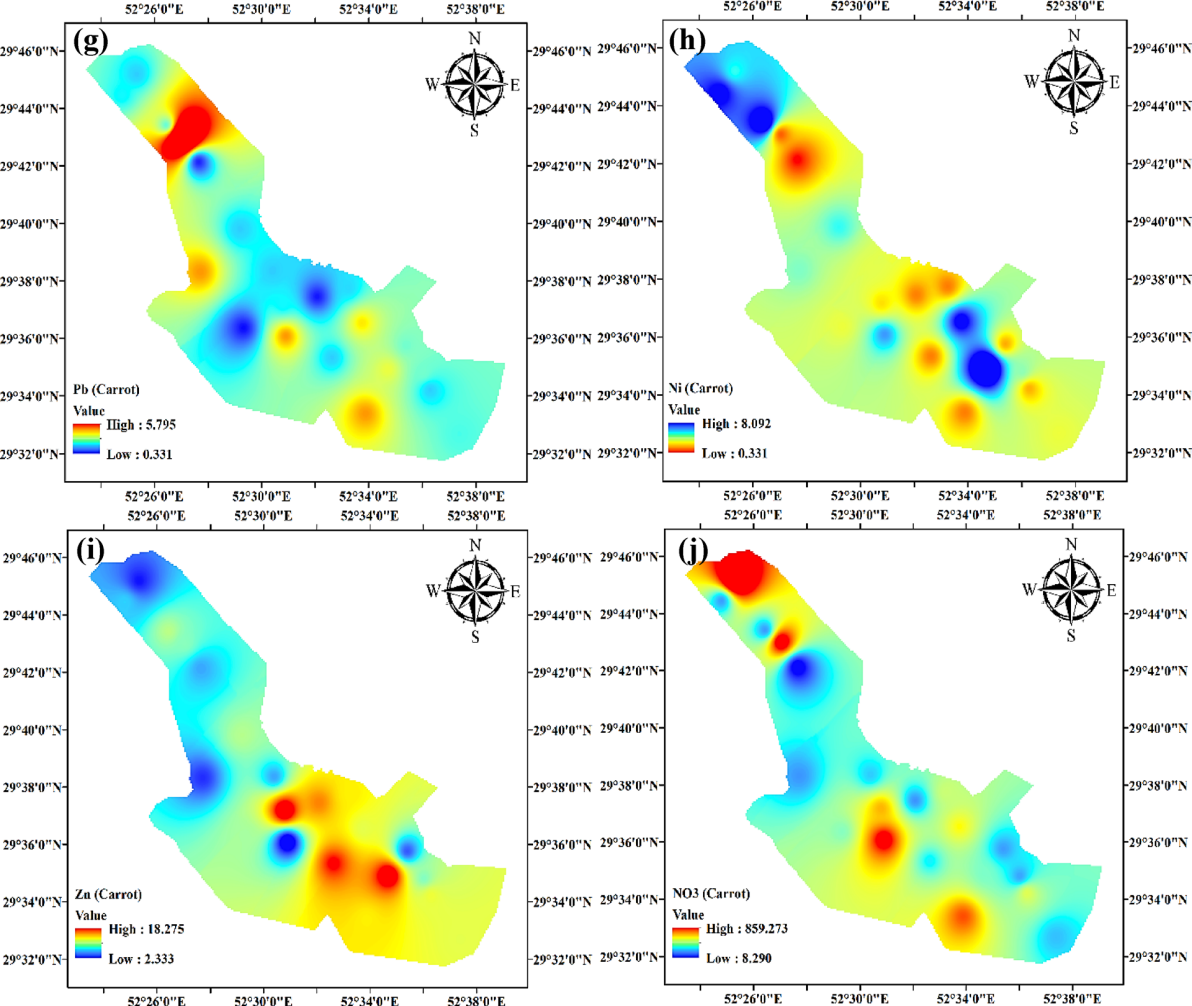
Regional distribution of metals (mg/kg) and nitrate (mg/L) for radish (**a-e**) and carrot (**f-j**).

The spatial distribution of Cd, Pb, Ni, Zn, and NO_3_ in radish and carrot samples revealed notable variations in space across the research region (Fig. 4a-j). The levels of Cd in radish and carrot samples showed significant variation, ranging from 0.151 mg/kg to 4.096 mg/kg. The levels of Cd in the central and southern areas were higher, perhaps due to industrial operations, the use of phosphate fertilizers, or past contamination episodes. The presence of high amounts of Cd in these areas requires immediate care owing to Cd’s significant toxicity and its propensity to induce renal damage, bone damage, and cancer with prolonged exposure. In contrast, the northern and northeastern regions had lower quantities of Cd, indicating a lower pollution level or efficient natural mechanisms that reduce its presence^72,73^. The lead concentrations exhibited significant variation, ranging from 0.171 mg/kg to 6.794 mg/kg. The northwest region had the highest levels of Pb, which are probably associated with industrial emissions, traffic pollution, or lead-based pesticides in agriculture. The health consequences of high levels of Pb are significant, especially impacting children’s brain development. The center and northeastern areas, which exhibited lower amounts of Pb, may experience this due to naturally occurring lower background levels or the successful deployment of pollution management measures^74,75^. The nickel concentrations in radish and carrot samples varied between 0.251 mg/kg and 8.092 mg/kg, with the most significant levels seen in the southern and central regions of the research area. The distribution of this implies that potential sources might include industrial discharges, metal plating, or fertilizers with a high nickel concentration. Exposure to nickel is recognized to induce allergic responses and has the potential to cause cancer. The lower amounts seen in the northern and southeastern areas may suggest less industrial activity or the implementation of effective environmental laws^76,77^.

The zinc levels varied considerably, ranging from 2.333 mg/kg to 18.275 mg/kg. The southern and southeastern areas had the most elevated amounts, which may be attributed to the excessive use of zinc fertilizers or industrial waste discharge. Although Zn is a necessary nutrient, an excessive intake may result in health problems such as abdominal pain, anemia, and interference with the absorption of other vital minerals. The decreased levels of Zn in the northwestern and northern areas might be attributed to reduced agricultural practices or stricter environmental regulations^78–80^. The concentrations of nitrates exhibited substantial variation, spanning from 7.588 mg/kg to 859.275 mg/kg. The NO_3_ levels in the northwestern, northern, and central areas were elevated, most likely due to agricultural runoff from nitrogen-based fertilizers. Elevated concentrations of nitrates in vegetables may lead to health hazards, including methemoglobinemia or “blue baby syndrome” in babies, as well as probable associations with several types of malignancies. The south and east areas, which exhibited reduced NO_3_ concentrations, might see improvements by implementing enhanced agricultural practices or using natural mitigation mechanisms^81,82^. The notable variation in the regional distribution of these pollutants emphasizes the need for targeted intervention measures. Immediate action is needed to address the health concerns associated with consuming contaminated vegetables in regions with high Cd, Pb, Ni, Zn, and NO_3_ levels. Possible strategies for reducing contamination levels include the implementation of phytoremediation, soil cleaning, and enforcing more stringent limits on industrial emissions and agricultural activities.

### Monte carlo simulation

#### Carcinogenic simulation

**Fig. 5:**
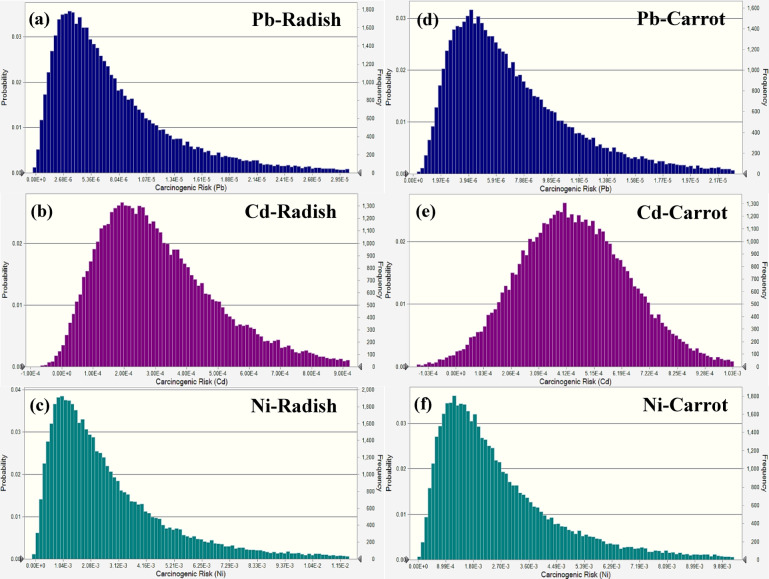
Carcinogenic risk simulation in radish (**a-c**) and carrot (**d-f**) for children.

The carcinogenic risk of Pb, Cd, and Ni exposure from radish and carrot consumption by children was assessed in this research using a probability-based simulation (Fig. 5). The findings were shown as probability distributions to illustrate the spectrum and likelihood of various danger levels. The average carcinogenic risk values for Pb, Cd, and Ni in radishes were around 2.52 × 10^−6^, 1.19 × 10^−4^, and 1.83 × 10^−3^, respectively, whereas the 95th percentile values for these metals were 3.78 × 10^−6^, 1.96 × 10^−4^, and 3.07 × 10^−3^, respectively. So, Cd and Ni results surpassed the recognized threshold of 1 × 10⁻⁴, indicating a possible danger to children’s health. The average carcinogenic risk for Cd and Ni in carrots was 2.07 × 10^−4^ and 3.12 × 10^−4^, respectively, with their 95th percentiles at 3.09 × 10^−4^ and 4.67 × 10^−4^, both over the tolerable carcinogenic risk level. Conversely, Pb levels in carrots, averaging 2.95 × 10^−6^ and reaching a 95th percentile of 4.85 × 10^−6^, indicated a far lower risk than the stated threshold, and were therefore deemed acceptable.

**Fig. 6:**
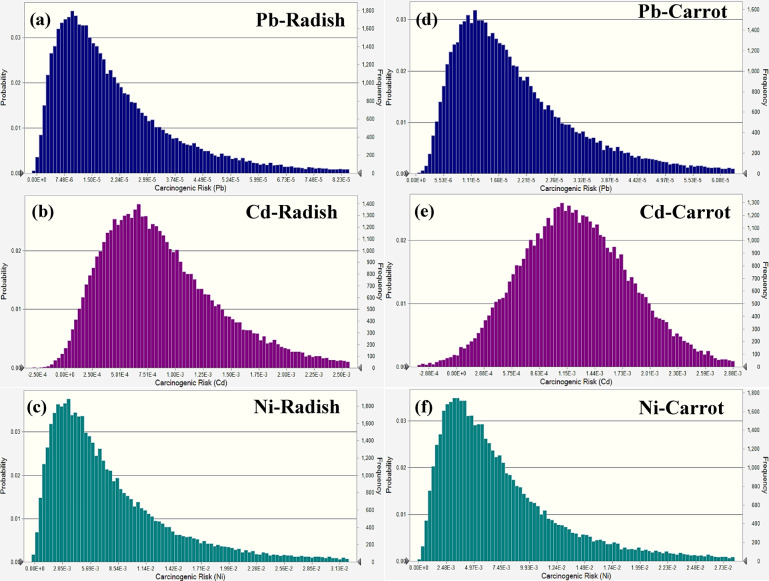
Carcinogenic risk simulation in radish (**a-c**) and carrot (**d-f**) for adult males.

In adult males (Fig. 6), the average carcinogenic risk values for radish concerning Pb, Cd, and Ni were 1.73 × 10^−6^, 8.17 × 10^−4^, and 1.26 × 10^−3^, respectively, with their 95th percentile values determined to be 2.59 × 10^−6^, 1.35 × 10^−4^, and 2.11 × 10^−3^, respectively. The findings suggested that the carcinogenic risk associated with Cd and Ni was above the acceptable threshold of 1 × 10^−4^, although Pb is near the threshold and is evaluated as a borderline danger. The average risk for Pb and Cd in carrots was 1.42 × 10^−5^ and 2.14 × 10^−4^, respectively, with their 95th percentiles at 2.12 × 10^−5^ and 3.20 × 10^−4^, both metals beyond the permissible risk limit. Nickel levels in carrots, averaging 2.03 × 10^−3^ and reaching a 95th percentile of 3.34 × 10^−3^, indicated a much-increased risk, maintaining considerably further the defined threshold and so categorized within the unacceptable range.

**Fig. 7:**
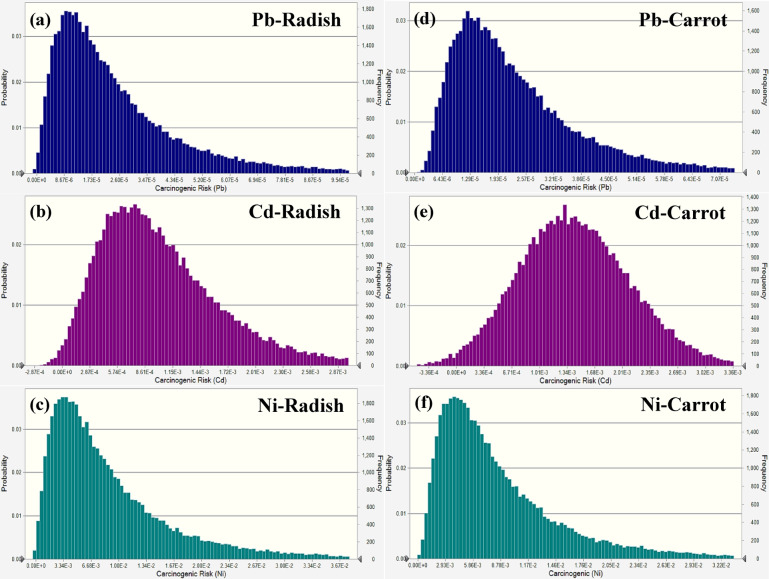
Carcinogenic risk simulation in radish (**a-c**) and carrot (**d-f**) for adult females.

The mean carcinogenic risk values for Pb, Cd, and Ni in adult females consuming radish were 1.94 × 10^−5^, 9.15 × 10^−4^, and 1.41 × 10^−3^, respectively, whereas the 95th percentile values were determined to be 2.91 × 10^−5^, 1.51 × 10^−4^, and 2.36 × 10^−3^, respectively. The findings demonstrated that the risks associated with Cd and Ni were beyond the established threshold of 1 × 10^−4^, posing a possible health danger to adult women, but Pb levels are near the threshold and are classified as a borderline risk. The average carcinogenic risk for Cd and Ni in carrots was 1.59 × 10^−4^ and 2.39 × 10^−3^, respectively, with their 95th percentiles at 2.38 × 10^−4^ and 3.57 × 10^−3^, both metals beyond the tolerable risk limit. Lead levels in carrots, averaging 2.28 × 10^−5^ and reaching a 95th percentile of 3.75 × 10^−5^, indicated a much-decreased risk, remaining far outside the stated threshold and therefore deemed acceptable (Fig. 7). These findings underscore that probabilistic analysis and concurrent monitoring of various heavy metals serve as a useful mechanism for detecting possible human health hazards and formulating risk management strategies.

#### Non-carcinogenic simulation

**Fig. 8:**
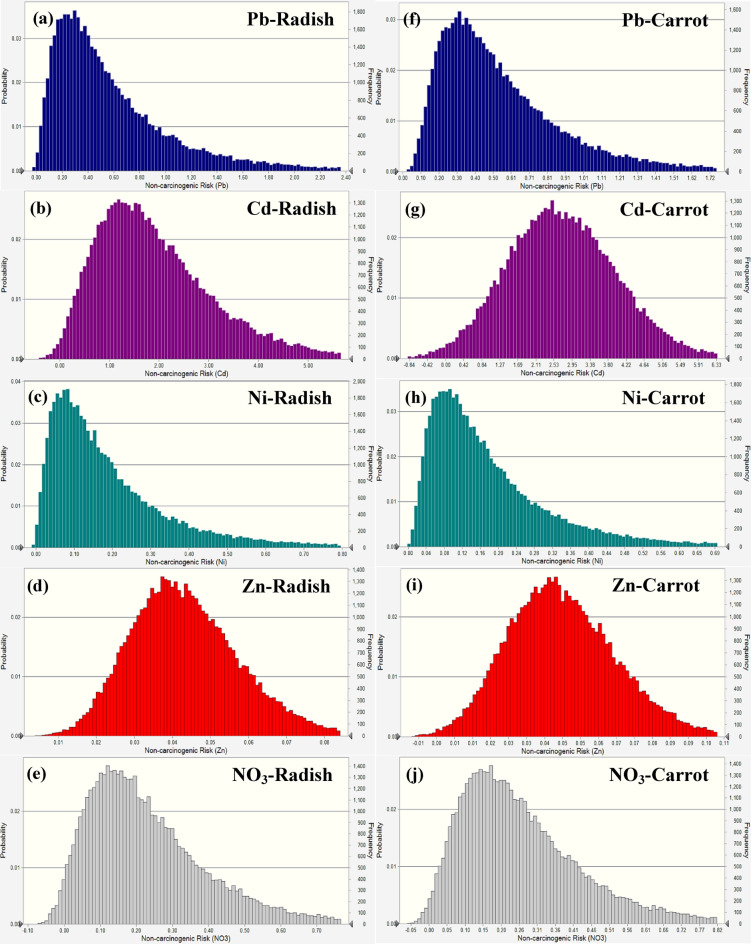
Non-carcinogenic risk simulation in radish (**a-e**) and carrot (**f-j**) for children.

A probabilistic analysis-based simulation was also used in this research to evaluate the non-carcinogenic health hazards associated with children eating carrots and radishes and being exposed to certain pollutants, such as Pb, Cd, Ni, Zn, and NO_3_ (Fig. 8). The average non-carcinogenic risk values for radishes were determined to be 0.98 for Pb, 1.39 for Cd, 0.61 for Ni, 0.18 for Zn, and 0.42 for NO_3_, with their respective 95th percentile values being 1.32, 1.63, 0.97, 0.29, and 0.68. The findings demonstrated that the risk from Cd was above the established threshold of 1.0, suggesting possible health issues, although Pb was near the threshold, and Ni, Zn, and NO_3_ stayed below safe ranges. The average risk for Pb, Cd, Ni, Zn, and NO_3_ in carrots was 0.86, 2.10, 0.79, 0.14, and 0.35, respectively, with their 95th percentile values determined to be 1.08, 0.16, 1.26, 0.22, and 0.56, respectively. In carrots, Cd above the permitted barrier, posing a considerable danger to children, although Pb, Ni, Zn, and NO_3_ stayed below permissible limits, with their associated risk deemed negligible.

**Fig. 9:**
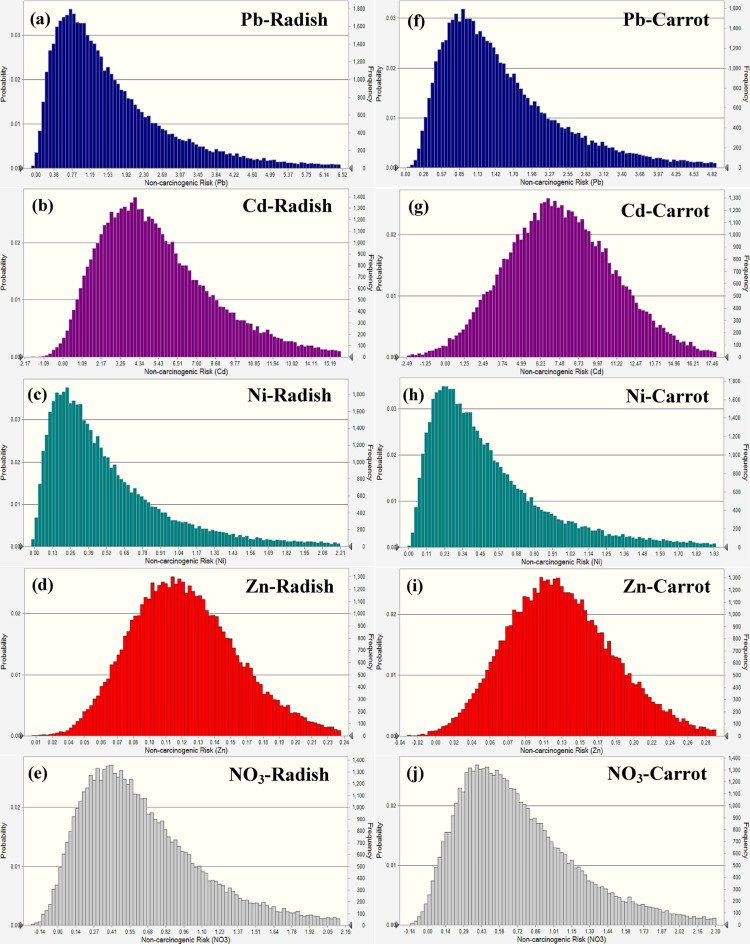
Non-carcinogenic risk simulation in radish (**a-e**) and carrot (**f-j**) for adult males.

In adult males, the mean HQ values for Pb, Cd, Ni, Zn, and NO_3_ in radish were determined to be 0.91, 2.27, 0.42, 0.12, and 0.31, respectively, with their 95th percentile values being 1.31, 2.44, 0.68, 0.19, and 0.51, respectively (Fig. 9). All values fell under the permissible threshold of 1.0 except Cd, indicating that the intake of radish contaminated with these components did not provide a substantial health risk to adult males. The mean HQ values for Pb, Cd, Ni, Zn, and NO_3_ in carrots were determined to be 0.72, 2.07, 0.54, 0.49, and 0.26, respectively, with their 95th percentile values being 0.64, 2.11, 0.36, 0.15, and 0.43, respectively. Like radish, all readings stayed below the permissible range except Cd, indicating safe exposure levels for adult males.

**Fig. 10:**
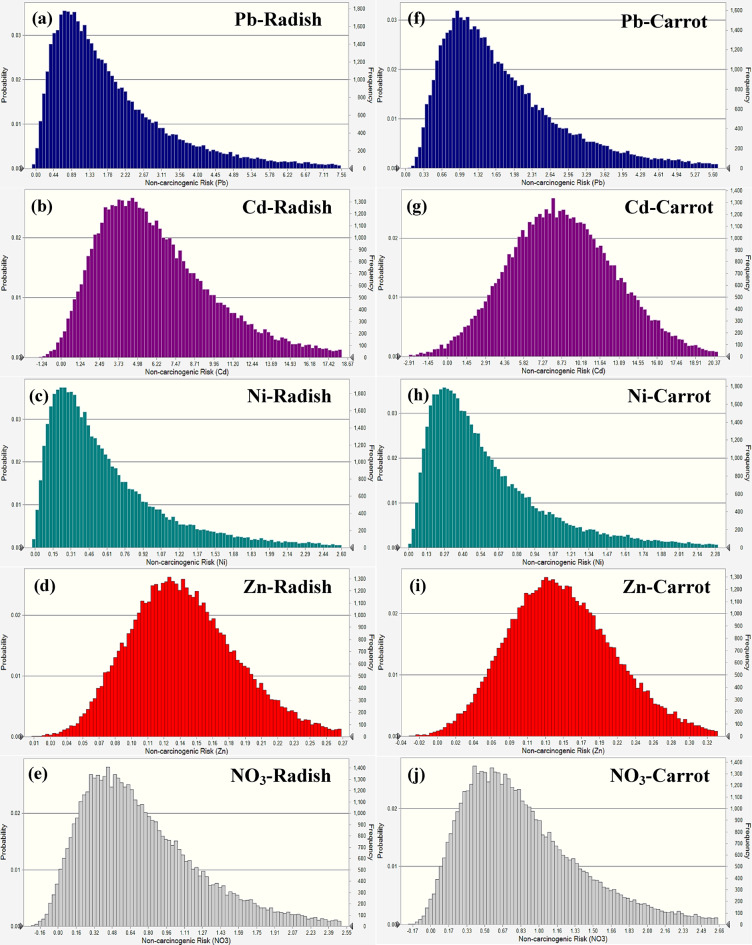
Non-carcinogenic risk simulation in radish (**a-e**) and carrot (**f-j**) for adult females.

Regarding radish, the mean HQ values for Pb, Cd, Ni, Zn, and NO_3_ in adult females were 0.97, 2.31, 0.48, 0.15, and 0.36, respectively (Fig. 10). The 95th percentile values for these elements were 1.52, 2.51, 0.78, 0.24, and 0.59. The findings demonstrated that Cd risk was above the recommended level of 1.0, presenting a possible health issue for adult women, but other aspects remained below permissible ranges. The mean HQ for Pb, Cd, Ni, Zn, and NO_3_ in carrots were 1.15, 2.78, 0.62, 0.11, and 0.29, respectively, with their 95th percentile values being 1.26, 2.13, 0.99, 0.18, and 0.47, respectively. In the carrot, Pb and Cd were above the safety barrier, and Ni neared the borderline danger level, although Zn and NO_3_ stayed within the acceptable range.

### Sensitivity analysis of HRA

#### Carcinogen sensitivity analysis

**Fig. 11:**
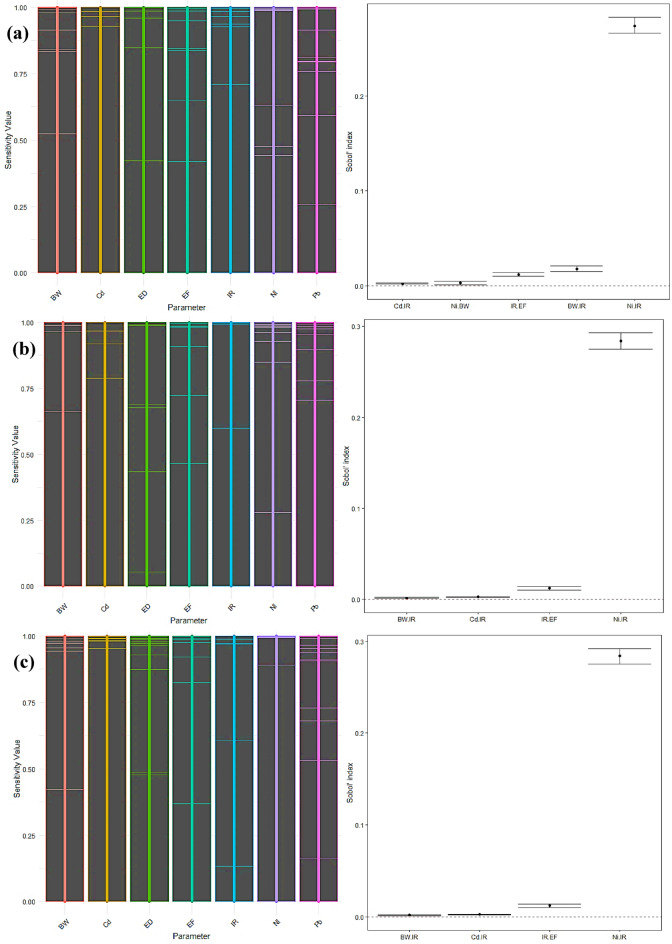
Sensitivity analysis for carcinogenic risk of radish in children (**a**), adult males (**b**), and adult females (**c**).

The *Sobol* sensitivity analysis evaluated the carcinogenic and non-carcinogenic hazards linked to the consumption of radish and carrot in three demographic groups: children, adult males, and adult females. The investigation concentrated on many crucial parameters, including cadmium (Cd), lead (Pb), nickel (Ni), nitrate (NO_3_), zinc (Zn), body weight (BW), intake rate (IR), exposure frequency (EF), and exposure duration (ED) (Fig. 11a-c). In children, the analysis of first-order sensitivity indices revealed that the IR had the highest influence on carcinogenic risk, with a value of 0.591. Ni had the second-highest impact, with a first-order effect of 0.074. Additional covariates, including Cd, BW, and EF, exhibited negligible individual impacts, as shown by first-order indices of 0.001, 0.005, and 0.003, respectively. The effect of Pb and ED was minimal, as indicated by an index of 0.000. The total effect indices, including individual effects and interactions, highlight the significance of IR (0.913) and Ni (0.367). The cumulative impact of BW (0.035) exceeded its primary impact, indicating potential interactions with other components, notably IR. Cd and EF had minor overall impacts of 0.003 and 0.022, respectively, whereas Pb and ED remained insignificant. The study found significant interactions between Cd-IR, Ni-BW, IR-EF, BW-IR, and Ni-IR. These interactions suggest that the combined effects of these factors play a role in the variation in cancer risk in children.

Among adult men, the primary impacts showed a consistent pattern, with IR having the highest influence (0.612) and Ni having a lesser influence (0.076). Cd, EF, and BW made insignificant contributions, with first-order indices of 0.001, 0.003, and 0.000, respectively. The effects of Pb and ED were negligible. The total impact indices for IR (0.918) and Ni (0.369) aligned with the findings in children, highlighting their significant contributions to the risk of developing cancer. The combined impacts of BW (0.002) and EF (0.022) saw a modest rise, suggesting the presence of interactions, notably with IR. The interactions among Cd-IR, IR-EF, BW-IR, and Ni-IR were notable, resembling the patterns reported in the children’s group. This indicates a continuous pattern of interaction across different age groups.

The sensitivity analysis revealed that IR had the highest significance level among adult females, with a first-order impact of 0.611. This was followed by Ni, which had a first-order effect of 0.076. The other covariates, Cd, EF, and BW, exhibited negligible individual effects, consistent with the observed trends in the different groups. The total impacts for IR (0.918) and Ni (0.369) were maintained at a high level, whereas Cd and EF showed little increase in their total effects (0.003 and 0.022, respectively), suggesting the presence of interactions. The analysis revealed a negligible overall impact (0.002) shown by BW, indicating few interactions. The research revealed noteworthy interactions among Cd-IR, IR-EF, BW-IR, and Ni-IR, corresponding to the interaction patterns seen in children and adult men.

The IR was shown to be the most critical factor in all groups, as indicated by both first-order and total effect indices. It plays a significant role in defining the carcinogenic risk. Ni was shown to be the second most influential component, exhibiting substantial overall effects greatly enhanced by interactions with IR. This emphasizes the need to control nickel exposure, especially in groups with elevated ingestion rates. Additional covariates, such as Cd, BW, and EF, had lesser direct impacts but contributed to overall effects owing to their interactions, notably with IR. This suggests that although these variables may have a lesser effect on their own, they might still add to the risk of developing cancer when paired with high rates of ingestion. The presence of Pb and the length of ED were consistently determined to be insignificant in all groups, indicating that they do not have a significant impact on the risk of developing cancer connected to the intake of radishes^83^. The detected interactions, especially those involving IR and Ni, emphasize the need for a complete risk assessment methodology that considers individual components and cumulative impacts. These interactions emphasize the intricate nature of cancer-causing risk and the need to take into account various factors^84^.

**Fig. 12:**
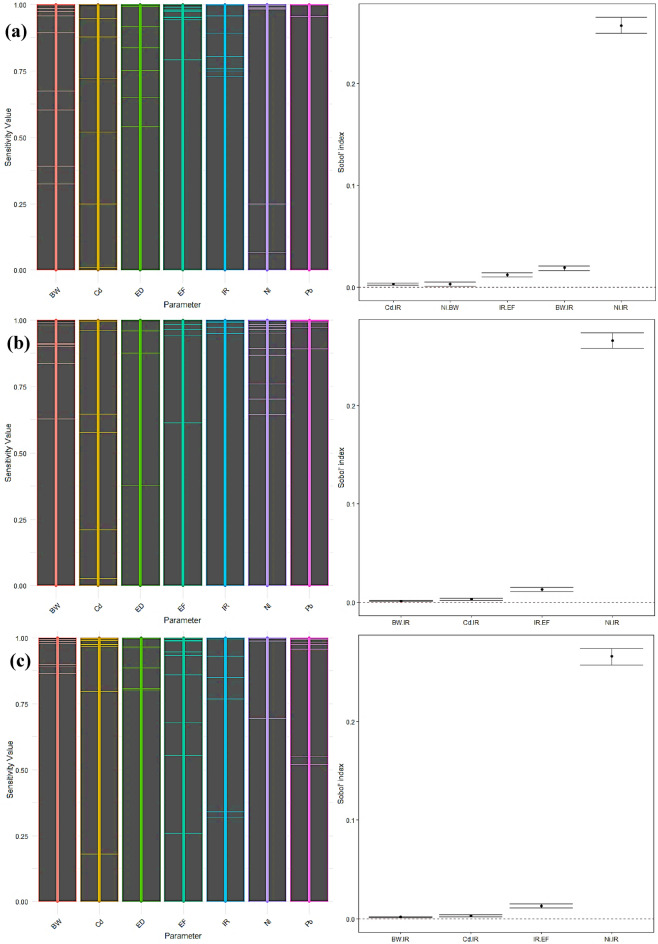
Sensitivity analysis for carcinogenic risk of carrot in children (**a**), adult males (**b**), and adult females (**c**).

According to the *Sobol* sensitivity analysis, the IR of carrots was the most critical factor in predicting carcinogenic risk in children, with a first-order sensitivity index of 0.612 (Fig. 12a-c). This suggests that IR plays a significant role in the total level of risk. Ni was determined to be the second most influential factor, with a first-order index of 0.069. The overall sensitivity indices confirmed the significant impact of IR, which had a total effect value of 0.917, and emphasized that Ni’s influence becomes more prominent when interactions are considered, with a total effect index of 0.344. In addition, the BW exhibited a total impact increase of 0.035, indicating its participation in interactions with other components. In contrast, the presence of Pb and the length of ED had little impact on the chance of developing cancer.

The research revealed that IR, with a first-order sensitivity index of 0.634 and Ni at 0.071, was the most critical factor in carrot consumption by adult men. The examination of total effects emphasized the substantial importance of IR, which had a total effect index of 0.923. On the other hand, Ni’s total impact of 0.345 indicated its noteworthy interactions with other components. The impact of BW was low when analyzed using a first-order approach. However, there was a tiny rise in the overall effect, reaching a value of 0.002, suggesting minor interactions. When studying youngsters, it was shown that both Pb and ED had little impact on the chance of developing cancer.

The sensitivity analysis for adult females exhibited comparable patterns to those reported in adult men. IR had the most significant influence in this context, with a primary impact of 0.633 and an overall impact of 0.922. Ni also exhibited a considerable influence, with primary and overall effects of 0.071 and 0.345, respectively. BW had a marginal rise in its overall impact, reaching 0.003, indicating a restricted level of interactions. The levels of Pb and the length of ED were minimal in all demographic categories.

#### Non-carcinogen sensitivity analysis

**Fig. 13:**
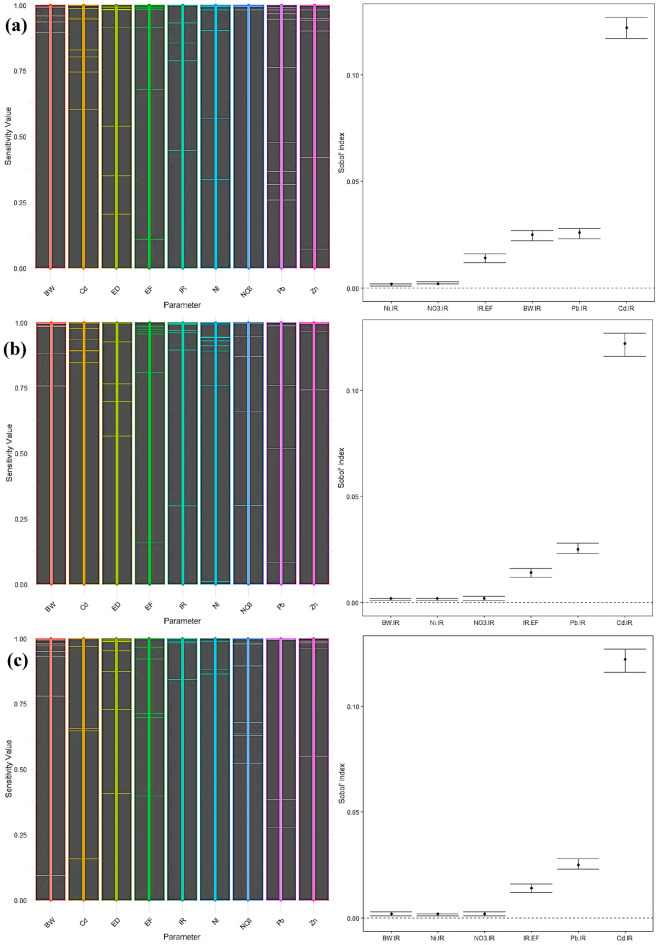
Sensitivity analysis for non-carcinogenic risk of radish in children (**a**), adult males (**b**), and adult females (**c**).

The sensitivity analysis results for children showed that, with a first-order sensitivity index of 0.785, IR was the most essential factor in predicting the non-carcinogenic risk associated with radish consumption (Fig. 13a-c). This significant impact emphasizes the crucial significance of IR in the comprehensive risk evaluation. Cd was determined to be the second most influential factor, with a first-order impact of 0.009. The overall sensitivity indices highlighted the predominance of IR, as shown by a total effect value of 0.984. When taking into account interactions, the impact of cadmium increased, resulting in a total effect of 0.139. The overall impact of BW increased to 0.032, indicating its participation in interactions with other parameters. The presence of Pb and NO_3_ had little effect on the non-carcinogenic risk. Notable correlations were observed between Ni and IR, NO_3_ and IR, IR and EF, BW and IR, Pb and IR, and Cd and IR, suggesting intricate interrelationships among these components.

The sensitivity analysis again showed IR as the most influential component among adult men, with a first-order sensitivity index of 0.782. Cd had a significant impact, with a first-order effect of 0.033. The comprehensive study demonstrated the prominent influence of IR, as shown by a total impact index of 0.952. The cumulative impact of cadmium was 0.159, indicating its heightened importance resulting from its interactions with other variables. Pb and NO_3_ had significant overall impacts of 0.034 and 0.003, respectively, highlighting their involvement in interactions with IR. The effect of BW was small when analyzed using a first-order approach, but it had a tiny increase in the overall effect, reaching a value of 0.003. Zn and Ni had little effect. Notable interactions were detected among Pb and IR, Cd and IR, IR and EF, BW and IR, Ni and IR, and NO_3_ and IR.

The sensitivity analysis in adult females exhibited comparable patterns to those in adult men. The IR was shown to be the most significant element, with a first-order impact of 0.781 and a total impact of 0.952. Cd had a first-order impact of 0.033 and a total effect of 0.159, indicating its substantial influence via interactions with other components. The cumulative effects of Pb and NO_3_ rose by 0.034 and 0.003, respectively. The overall impact of BW showed a slight rise of 0.003. Zn and Ni made little contributions to the non-carcinogenic risk. Significant connections were seen between Pb and IR, Cd and IR, IR and EF, BW and IR, Ni and IR, and NO_3_ and IR.

The significant first-order and total sensitivity indices for IR across all groups highlight its crucial contribution to assessing non-carcinogenic risk. Efficiently managing IR is essential for minimizing this risk. Cd has been identified as a significant factor, with its influence being particularly pronounced in the overall effects study as opposed to the primary effects. This implies that while Cd has a relatively little direct impact, its influence becomes more significant when considering interactions with other components. BW, Pb, and NO_3_ showed elevated overall effects, suggesting their participation in substantial interactions with IR. The study revealed noteworthy connections between Cd-IR, Pb-IR, and IR-EF, highlighting the intricate interplay among these components. The elements Ni and Zn consistently had little effect on non-carcinogenic risk, indicating that they have a relatively low influence on radish intake. The ED exhibited negligible impacts on all demographic categories^85^.

**Fig. 14:**
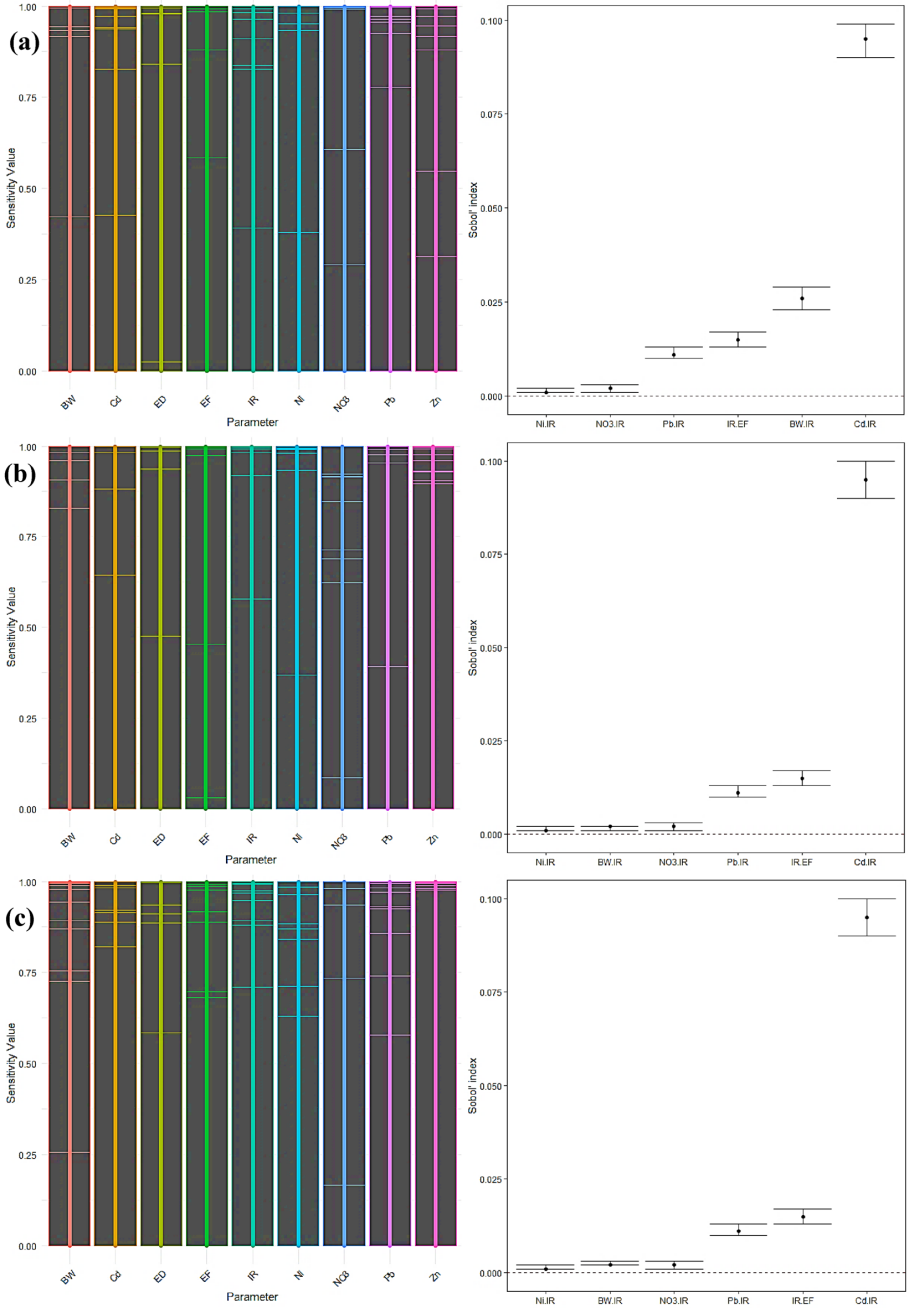
Sensitivity analysis for non-carcinogenic risk of carrot in children (**a**), adult males (**b**), and adult females (**c**).

According to the children’s carrot consumption sensitivity study, the most significant component was found to be IR, which had a first-order sensitivity index of 0.831 (Fig. 14a-c). Cd had the second -highest impact, with a first-order effect of 0.007. The overall sensitivity indices confirmed the significance of IR, with a total effect value of 0.988. The overall impact of cadmium rose to 0.107, demonstrating its essential involvement in influencing other components via interactions. The overall effect of BW increased to 0.032, indicating its role in interactions. The impact of Pb and NO_3_ on non-carcinogenic risk was negligible. Notable interactions were detected between Ni and IR, NO_3_ and IR, IR and EF, BW and IR, Pb and IR, and Cd and IR. These interactions demonstrate the intricate connections between several variables that influence non-carcinogenic risk.

The IR had the highest influence on adult men, with a first-order sensitivity index of 0.834. Cd had the second-highest impact, with a first-order effect of 0.026. The comprehensive study demonstrated the significant influence of IR, as shown by a total impact index of 0.964. The overall impact of cadmium was 0.124, indicating its heightened importance when interactions are considered. The elements Pb and NO_3_ had significant overall impacts of 0.015 and 0.003, respectively. The influence of BW was minor in terms of a first-order effect, but it contributed somewhat to a total effect of 0.003. Zn and Ni had insignificant impacts on non-carcinogenic risk. Significant interactions were seen between Pb and IR, Cd and IR, IR and EF, BW and IR, Ni and IR, and NO_3_ and IR.

Among adult females, the IR was shown to be the most crucial element, with a first-order impact of 0.834 and a total effect of 0.963. Cd had a first-order impact of 0.026 and a total effect of 0.124, indicating its substantial contribution via interactions. The cumulative impacts of Pb and NO_3_ rose by 0.015 and 0.003, respectively. The overall effect of BW showed a slight rise of 0.003. Zn and Ni made little contribution to the non-carcinogenic risk. Notable interactions were detected among Pb and IR, Cd and IR, IR and EF, BW and IR, Ni and IR, and NO_3_ and IR.

Cd has been identified as a significant component, with its influence being more pronounced in the total effects analysis than the first-order effects. This implies that while Cd has a relatively little direct impact, its influence becomes more prominent when considering interactions with other components. BW, Pb, and NO_3_ exhibited elevated overall effects, suggesting their involvement in significant interactions with IR. The notable correlations among Pb-IR, Cd-IR, and IR-EF indicate the intricate interconnections among these variables. Ni and Zn consistently show little effect on non-carcinogenic risk, suggesting their influence on carrot intake is limited. The ED exhibited negligible impacts on all groups.

## Conclusion

This research sheds light on the potential carcinogenic and non-carcinogenic dangers of eating carrots and radishes, especially when it comes to heavy metal poisoning. Children consistently exhibited higher HQ values compared to adult men and females, which may be attributed to their lower body weight, increasing their susceptibility to pollutants. Cadmium (Cd) was determined to be the primary factor contributing to non-carcinogenic risk, displaying the highest HQ values. This aligns with Cd’s established toxicological profile, whereby it has a propensity to accumulate in critical organs such as the kidneys, lungs, and liver, even when exposed to low levels. On the other hand, Zn had the lowest HQ values, indicating a comparatively reduced level of risk. The research evaluated both non-carcinogenic hazards and carcinogenic risks linked to the intake of these vegetables. The carcinogenic risk values for Cd and Pb were consistently lower than the permitted risk threshold set by the USEPA. This indicates no significant concerns about the potential for cancer development in the evaluated populations. Nevertheless, Ni showed a slightly higher risk of causing cancer(in comparison to Pb and Cd), especially in adolescents, though cancer risk values were slightly over the risk threshold advised by the USEPA (1 × 10^−4^). This discovery was worrisome since extended exposure to even small amounts of Ni might heighten the probability of cancer formation. The *Sobol* sensitivity study highlighted the significance of Cd, especially concerning parameters such as IR and BW. Additionally, interactions between Pb and NO_3_ were detected, although to a lower degree. The geographical study of heavy metal distribution demonstrated notable heterogeneity, with elevated Cd, Pb, and Ni levels in some areas, presumably attributable to industrial operations, agricultural practices, and environmental variables. This variance highlights the need to implement appropriate measures at each location to reduce the health hazards of consuming vegetables contaminated with heavy metals. In general, while eating carrots and radishes poses considerable non-carcinogenic hazards, mainly owing to the presence of Cd, it is crucial not to disregard the carcinogenic dangers, mainly from Ni. To mitigate these hazards, it is critical to implement effective risk management measures, such as implementing more stringent regulations on agricultural inputs and implementing specific public health initiatives. Public health strategies should prioritize the reduction of heavy metal exposure in food, with particular emphasis on safeguarding vulnerable groups, such as children, who are more susceptible to both non-carcinogenic and carcinogenic impacts.

## Data Availability

Data will be made available on reasonable request.
